# The Analysis of Intracellular and Intercellular Calcium Signaling in Human Anterior Lens Capsule Epithelial Cells with Regard to Different Types and Stages of the Cataract

**DOI:** 10.1371/journal.pone.0143781

**Published:** 2015-12-04

**Authors:** Marko Gosak, Rene Markovič, Aleš Fajmut, Marko Marhl, Marko Hawlina, Sofija Andjelić

**Affiliations:** 1 Institute of Physiology, Faculty of Medicine, University of Maribor, Slovenia; 2 Faculty of Natural Sciences and Mathematics, University of Maribor, Maribor, Slovenia; 3 Faculty of Education, University of Maribor, Maribor, Slovenia; 4 Faculty of Health Sciences, University of Maribor, Maribor, Slovenia; 5 Eye Hospital, University Medical Centre, Ljubljana, Slovenia; University of Delaware, UNITED STATES

## Abstract

In this work we investigated how modifications of the Ca^2+^ homeostasis in anterior lens epithelial cells (LECs) are associated with different types of cataract (cortical or nuclear) and how the progression of the cataract (mild or moderate) affects the Ca^2+^ signaling. We systematically analyzed different aspects of intra- and inter-cellular Ca^2+^ signaling in the human LECs, which are attached to surgically isolated lens capsule (LC), obtained during cataract surgery. We monitored the temporal and spatial changes in intracellular Ca^2+^ concentration after stimulation with acetylcholine by means of Fura-2 fluorescence captured with an inverted microscope. In our analysis we compared the features of Ca^2+^ signals in individual cells, synchronized activations, spatio-temporal grouping and the nature of intercellular communication between LECs. The latter was assessed by using the methodologies of the complex network theory. Our results point out that at the level of individual cells there are no significant differences when comparing the features of the signals with regard either to the type or the stage of the cataract. On the other hand, noticeable differences are observed at the multicellular level, despite inter-capsule variability. LCs associated with more developed cataracts were found to exhibit a slower collective response to stimulation, a less pronounced spatio-temporal clustering of LECs with similar signaling characteristics. The reconstructed intercellular networks were found to be sparser and more segregated than in LCs associated with mild cataracts. Moreover, we show that spontaneously active LECs often operate in localized groups with quite well aligned Ca^2+^ activity. The presence of spontaneous activity was also found to affect the stimulated Ca^2+^ responses of individual cells. Our findings indicate that the cataract progression entails the impairment of intercellular signaling thereby suggesting the functional importance of altered Ca^2+^ signaling of LECs in cataractogenesis.

## Introduction

The function of the lens, which is a transparent organ suspended between the aqueous humor and the vitreous, is to transmit and focus light on the retina. Cataracts are opacities of the lens and are the leading cause of blindness worldwide with the 37 million people affected, 48% of world blindness [1]. Several lines of evidence implicate the loss of Ca^2+^ homeostasis in the lens as a key factor in cataract formation [2]. Although many studies have found a correlation between elevated total lens Ca^2+^, free+bound, and cataract (for review see [3]), a clear disambiguation between the cause and effect relationship has not been found. Ca^2+^ is a universal intracellular messenger involved in essential cellular functions and it is a key mediator of signaling within lens cells. The cataract is a result of the functional impairment of both constitutive types of lens cells, LECs that form a single layer along the anterior surface of the lens, and fiber cells that form the bulk of the lens. LECs are metabolically the most active part of the lens, acting as the metabolic engine that sustains the physiological health of the lens. They regulate most of the homeostatic functions of the lens since they contain most mechanisms of metabolism, synthesis and active transport [4]. However, the role of LECs in controlling the lenticular Ca^2+^ is not completely understood. It is observed that irrespective of the type of cataract, total Ca^2+^ levels are always several-fold higher in the LECs from the central zone of epithelium of the lenses with the cataract than in those taken from the clear controls. However, it seems that there are no significant differences between the two most frequently present types of cataract, cortical, C, and nuclear, N, cataracts [5]. Free cytoplasmic Ca^2+^ concentration, [Ca^2+^]_i_, in LECs is always kept low under physiological conditions. The duration and the magnitude of [Ca^2+^]_i_ elevation is generally very tightly regulated. Entry of Ca^2+^ into the LECs is highly regulated by different receptors [6]. The first Ca^2+^ signaling agonist to be identified in the lens was ACh [7]. LECs respond with a rise in [Ca^2+^]_i_ [7–9] after its application. In human anterior LECs ACh binds to M1 muscarinic receptors inducing a rise in [Ca^2+^]_i_ [6,8,10].

An important question in LECs research is the role of the altered intra- as well as inter- cellular signaling (including Ca^2+^ signaling) in LECs and the subsequent effect this may have in cataract formation [11–13]. Intercellular communication mediated by gap junction channels has been proposed to have a major role in the maintenance of lens transparency [14], and the important role of LECs´ gap junction channels have been shown [15]. Gap junction channels facilitate transfer of ions and molecules up to 1 kDa among coupled cells [11,16]. From the mathematical point of view, cytoplasm of adjacent individual LECs, which are interconnected by gap junctions, could be treated as interconnected autonomous dynamical systems. As such, they can be studied by means of graph-theoretical approaches, whereby the cells represent the nodes in a network and the links signify the intercellular interactions. In the last years, the modern network theory has become a cornerstone for the description and the analysis of interactions within diverse complex systems [17,18]. Notably, this methodological concept has proven to be very useful for the quantification of organizational and functional principles of living organisms across various scales. Non-trivial and similar topological interaction patterns, characterized with the small-world property, a heterogeneous degree distribution and/or a modular structure, have been found and successfully evaluated within a plethora of biological systems, such as the intracellular metabolic and molecular interaction networks [19,20], composition of microbial communities [21], relations between physiologic systems [22], and the brain’s functional and anatomical organization [23]. With the advances of live cell imaging techniques that facilitate non-invasive recordings of cellular dynamics in several cells simultaneously, the network analysis has also been successfully applied for studying collective dynamics of cell populations at the tissue level [24–31]. In the present report we follow these ideas and extend the application of complex network methodology for the assessment of the intercellular connectivity patterns and Ca^2+^ signal propagation between LECs in the human LC preparation by means of statistical characterization of the extracted networks of LECs.

In our previous studies, we have already shown that the human anterior LC preparation is an adequate source for investigating cellular Ca^2+^ dynamics of LECs in different cataract types. The methodology of cellular Ca^2+^ dynamics measurements of LECs was presented in detail in Ref. [32]. We have studied the contractions of anterior LC LECs and their association with the Ca^2+^ dynamics of the cells [33]. Furthermore, we have studied the anterior LECs functionality upon gentian violet staining of the anterior LC during cataract surgery and found no influence on the cell functionality and Ca^2+^ dynamics [34]. We have reviewed the topic of cataractogenesis [35], the role of Ca^2+^ in cataract formation [36] as well as the structural and functional characteristics of lens epithelium [36].

The aim of this study is to investigate Ca^2+^ signaling in LECs of different types of cataracts (C and N) as well as of different degrees of cataract progression (mild and moderate) in order to understand the connection between the altered Ca^2+^ homeostasis in individual LECs, intercellular communication between LECs and the cataract. The remainder of the paper is organized as follows. We first analyze the features of [Ca^2+^]_i_ dynamics in individual cells, giving special attention to the role of spontaneous activity in LECs. Next we focus on the collective behavior of LECs and examine the global responsiveness to stimulation, synchronization behavior and the spatio-temporal grouping of LECs. Finally, we assess the nature of intercellular communication by means of graph-theoretical approaches. Our results show that at the level of individual LECs there are no significant differences when comparing the features of Ca^2+^ signaling with regard either to the type or the stage of the cataract. On the contrary, a less orchestrated intercellular Ca^2+^ signaling between LECs associated with more developed cataracts is identified. We discuss our findings in the context of the role of intercellular communication for ensuring a normal functioning of multicellular systems and how its malfunctioning is associated with different pathologies.

## Results

We systematically analyzed different aspects of intra- and inter-cellular Ca^2+^ signaling in the human anterior lens epithelium, build from LECs, whereby we compare different signaling characteristics with regard to the type of the cataract–C and N, and the degree of cataract progression. The latter encompasses the comparison between mild, C&N, and moderate to severe, C2-4&N2-4, cataracts, irrespective of the type of cataract. In our analysis we analyze altogether 12 different C (2619 cells) and 13 different N cataract (2963 cells) LCs. In the group of mild, C&N, and moderate to severe, C2-4&N2-4, cataract LCs we have 15 (3964 cells) and 10 (1618 cells) samples, respectively. The experimental protocol consisted of the time before stimulation, the time of stimulation with ACh, and the subsequent recovery time.

**Fig 1:**
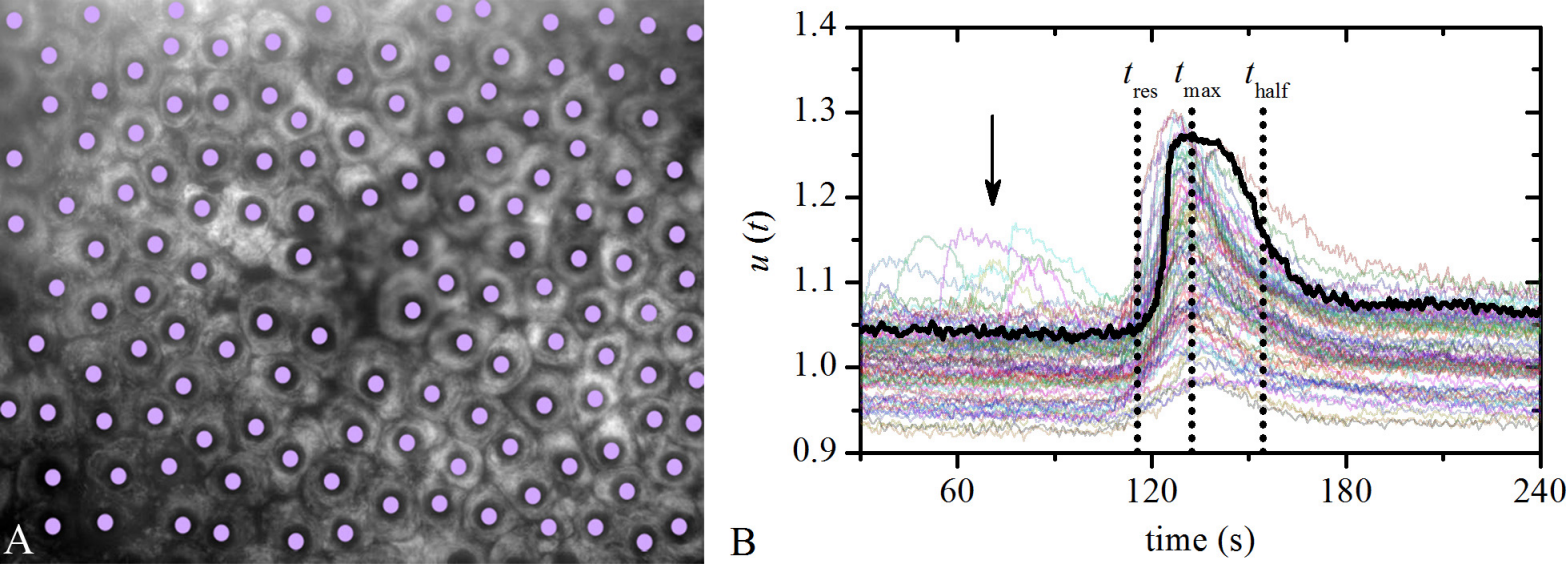
Measuring and analyzing LECs activity. **A** Typical image of the anterior LC epithelium recorded with 480 nm. Purple dots denote the midpoints of the ROIs of individual LECs. **B** Typical time traces of the intracellular Ca^2+^ dynamics in LECs, *u*(*t*) (ratio 360/380) in response to ACh. Relevant times of interest for a particular LEC are indicated with dotted vertical lines: *t*_res_, *t*_max_ and *t*_half_. The black arrow points out spontaneous activity.

In Fig 1A we show an image of a typical anterior LC’s epithelium with indicated centers of LECs (midpoints of regions of interest, i.e. ROIs). Each LEC responded to ACh stimulation with an increase in the [Ca^2+^]_i_, which was followed by a decrease towards the stationary state, as exemplified in Fig 1B. All cells did not respond to ACh stimulation at the same time. For the characterization of the intracellular Ca^2+^ signals we defined three parameters of the temporal characteristics of the signal: *t*_res_−the time at which a particular cell started to respond with a rise in [Ca^2+^]_i_ after stimulation with ACh; *t*_max_−the time at which the highest [Ca^2+^]_i_ of a particular cell was achieved; *t*_half_−the time at which [Ca^2+^]_i_ of a particular cell decreased to 50% of its maximal value (see Materials and Methods). These specific parameters enabled the quantification of the Ca^2+^ signal in each individual cell within the sample and represented the basis for all analyses of the intra- and inter- cellular signaling in the lens epithelium. Furthermore, some LECs exhibited spontaneous Ca^2+^ activity in the phase before the stimulation (corresponding [Ca^2+^]_i_ traces are indicated by an arrow in Fig 1B).

### Analysis of the temporal profiles of [Ca2+]i changes in LECs

**Fig 2:**
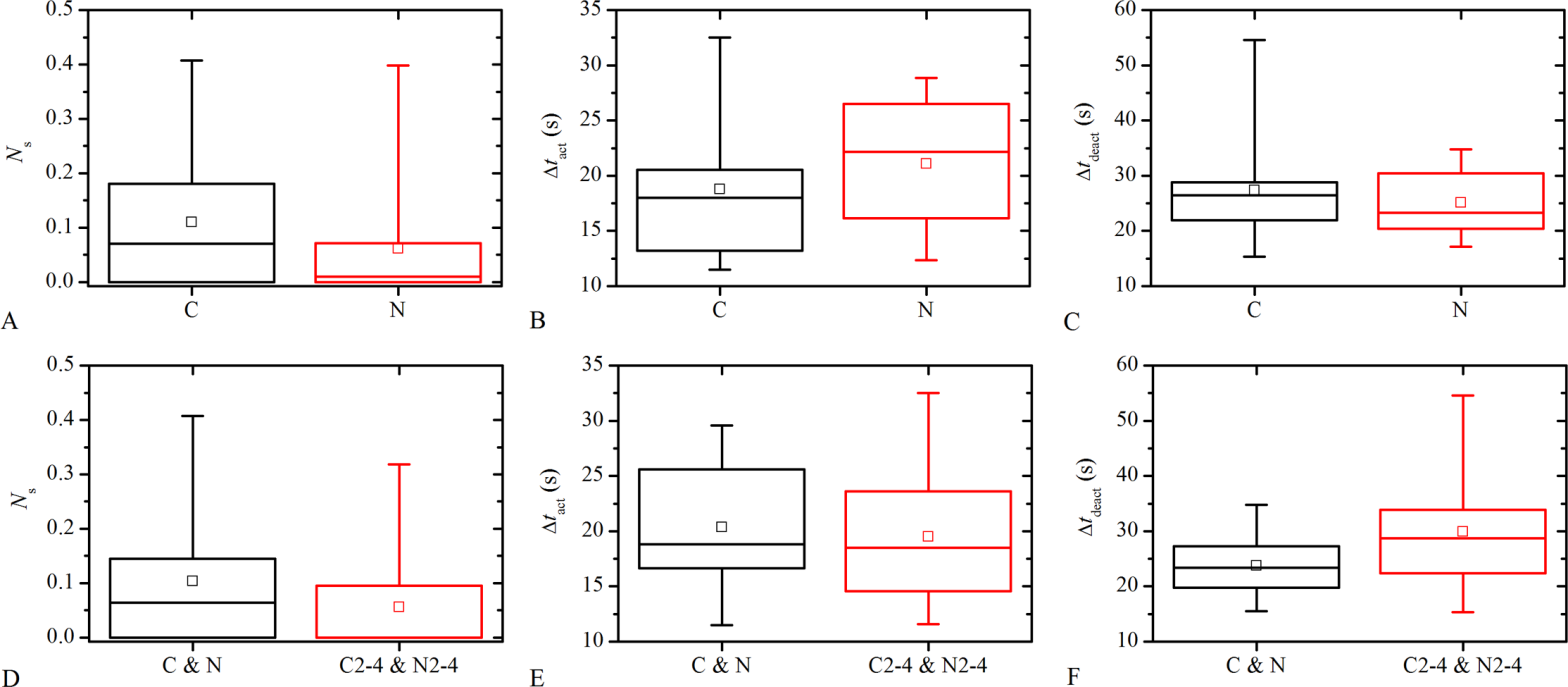
Analysis of the intracellular Ca^2+^ signaling in the human anterior LC epithelium. **A-C** The comparisons of the signaling characteristics (fraction of spontaneously active cells, *N*_S_, average activation time, Δ*t*_act_ = *t*_max_ − *t*_res_, and the average deactivation time, Δ*t*_deact_ = *t*_half_ − *t*_max_) with respect to the type of the cataract (C or N). **D-F** The same analysis as in A-C but with respect to the degree of the cataract (C&N or C2-4&N2-4). Values within individual groups are represented by means of the box charts diagrams in which boxes determinate the interval within 25th and 75th percentiles, whiskers denote the minimal and the maximal values, lines within the boxes indicate the median, and small squares stand for the average value. The *post hoc* one-way ANOVA statistical test has not identified statistically significant differences between any of the compared pairs.

Here we analyze temporal characteristics of intracellular Ca^2+^ signaling of each individual cell before and after ACh stimulation. With respect to the type and the degree of the cataract we compare three different variables: the fraction of spontaneously active cells, *N*_*S*_, Fig 2A and 2D, the average activation time, Δ*t*_act_ = *t*_max_ − *t*_res_, Fig 2B and 2E and the average deactivation time, Δ*t*_deact_ = *t*_half_ − *t*_max_, Fig 2C and 2F. Results in Fig 2 indicate that characteristics of intracellular signaling exhibit very high level of variability, irrespective of the type or the degree of the cataract. In 14 from all 25 samples, (56%), spontaneously active cells were detected, whereby the fraction of spontaneously active cells in each sample ranged from 1% to 40%. The average activation times, Δ*t*_act_, were in the range from 12 s to 32 s, and the average deactivation times, Δ*t*_deact_, were between 15 s and 54 s. To assess the possible differences between C and N cataracts as well as between C&N and C2-4&N2-4, we use the *post hoc* one-way ANOVA statistical test (see Materials and Methods). We couldn’t detected any significant differences with respect to the type and the degree of the cataract when comparing the fraction of spontaneously active cells, *N*_S_, the average activation time, Δ*t*_act_, and the average deactivation time, Δ*t*_deact_.

**Fig 3:**
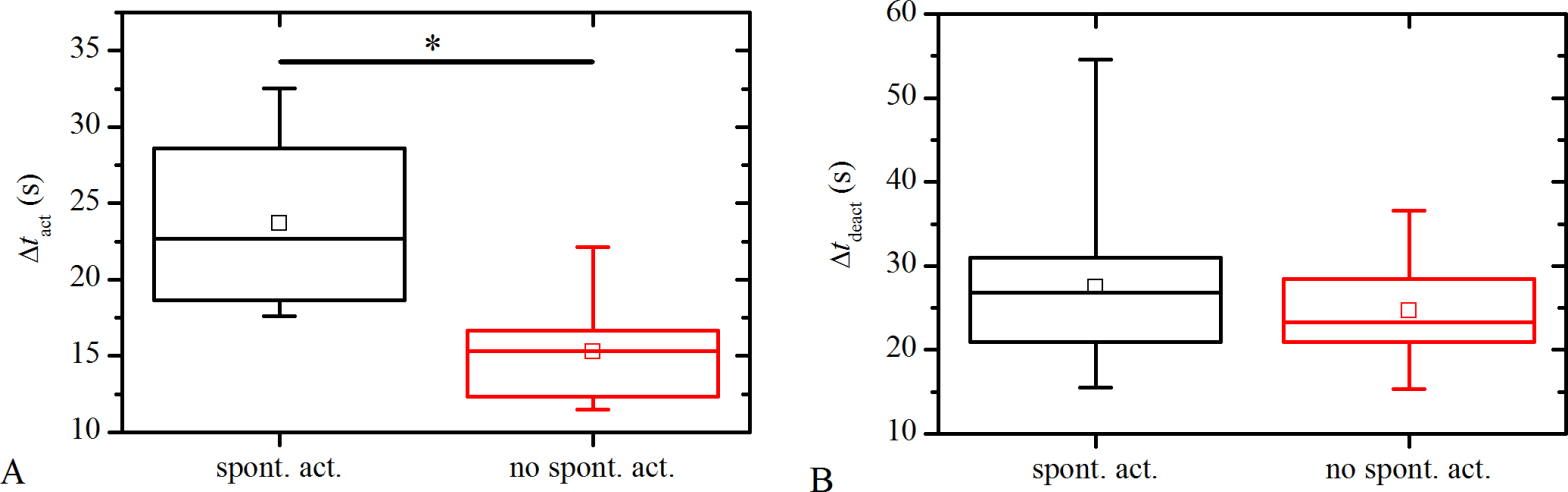
Comparison of the intracellular Ca^2+^ signaling characteristics between LCs with and without spontaneously active LECs. **A** Distribution of the average activation times, Δ*t*_act_, in the LCs with respect to the spontaneous activity. **B** The impact of the spontaneous activity on the average deactivation times, Δ*t*_deact_. One-way ANOVA statistical test have recognized statistically significant differences for Δ*t*_act_ with regard to the spontaneous activity (indicated by asterisk), whereas no significant differences could be detected by comparing deactivation times, Δ*t*_deact_. Box charts are defined the same as in Fig 2.

Next we examine how the presence of spontaneous LEC`s activity impacts the nature of [Ca^2+^]_i_ signals after ACh stimulation. For this purpose we compare the average activation times, Δ*t*_act_, and the average deactivation times, Δ*t*_deact_, of LCs that do and do not have spontaneously active cells, irrespective of the type or the degree of the cataract. Results presented in Fig 3A reveal that LCs in which spontaneously active LECs are present, have on average higher activation times, Δ*t*_act_. The differences are statistically significant, as confirmed by the ANOVA test. On the other hand, the presence of spontaneous activity does not seem to significantly affect the average deactivation times (Fig 3B). These results indicate that spontaneous activity slows down the activation part of the [Ca^2+^]_i_ transient provoked by ACh, whereas it does not have a considerable effect on the deactivation part. To get additional insights into the phenomenon, we compare the activation and deactivation times of all cells with respect to spontaneous activity. To overpass the inter-capsule variability in the average signaling times (see Fig 2), individual times, Δ*t*_act,*i*_ and Δ*t*_deact,*i*_, were divided by the average time of the given LC. Results presented in S1 Fig reveal that individual spontaneously active cells have in average higher activation times in comparison to spontaneously non-active cells. On the other hand, statistically significant differences could not be detected by comparing individual deactivation times in this case.

### Characterization of correlations in [Ca^2+^]_i_ dynamics of LECs

**Fig 4:**
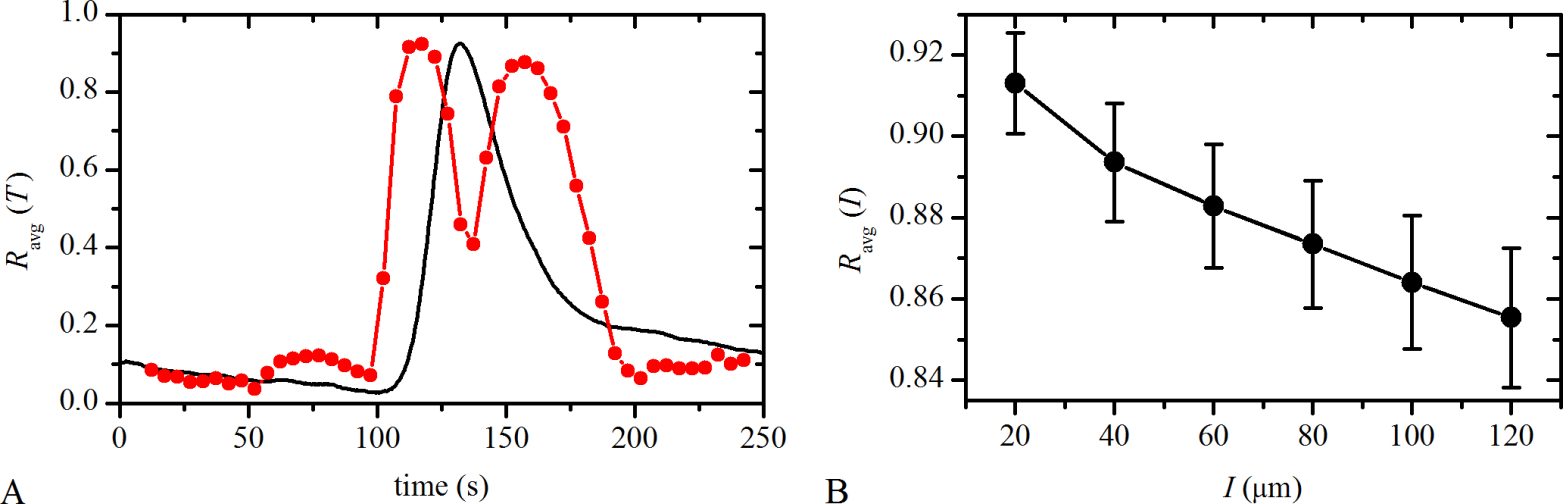
Correlation of Ca^2+^ dynamics in LECs. **A** The average correlation coefficient, *R*_avg_(*T*), as a function of time (red line with dots) and the corresponding average signal of all LECs in a typical cortical LC (black line). **B** The average correlation coefficient, *R*_avg_(*I*), as a function of the Euclidean distance, *I*, between LECs. Individual points represent the average correlation of all cell pairs within a given interval throughout the whole time series. All points were obtained by averaging over all LCs (25 capsules, 5653 cells). The error bars denote the corresponding standard errors.

For the quantification of the temporal evolution of correlations in Ca^2+^ dynamics between LECs we use the sliding window correlation analysis (see Materials and Methods). In particular, we calculate the average correlation coefficient between all pairs of LECs, *R*_avg_(*T*), within a given small time-interval that was being slid along the recorded time series. Values of *R*_avg_(*T*) close to 0 indicate rather low correlations between [Ca^2+^]_i_ signals in LECs at the given point in time, whereas on the other hand, values close to 1 signify well synchronized dynamics. Results in Fig 4A show the average correlation coefficient, *R*_avg_(*T*), as a function of time (red line with dots), and the corresponding average signal of all LECs (black line). It can be observed that the level of correlation between LECs either prior to or after the response to ACh is rather low. On the other hand, within the activation/deactivation phases *R*_avg_(*T*) is very high. This is a result of rather simultaneous increases/decreases of [Ca^2+^]_i_ in all LECs. The decrease in *R*_avg_(*T*) in the region of maximal ACh-induced [Ca^2+^]_i_ is attributed to the time-delays between individual signals. In other words, while in some cells [Ca^2+^]_i_ begins to decrease, in other cells, which probably responded to Ach stimulation with a delay, [Ca^2+^]_i_ is still increasing. Hence, in this situation average correlation is lower. Furthermore, small increases of *R*_avg_(*T*) in the period prior to Ach-induced [Ca^2+^]_i_ transients (between 50 s and 100 s) are a consequence of spontaneous activity in the locally synchronized clusters of LECs. Namely, spontaneously active LECs are frequently found to operate in localized groups with a concurrent [Ca^2+^]_i_ activity, as exemplified in S2 Fig. It should be noted that in all LCs with spontaneously active cells a conceptually very similar behavior is observed.

For a more detailed insight into the synchronization behavior we calculate the average correlation as a function of physical distances, *R*_avg_(*I*), whereby the average correlation between all LECs at a given distance is calculated over the whole recorded time series of the [Ca^2+^]_i_ activity. The results in Fig 4B reveal that *R*_avg_(*I*) is monotonically decreasing function of distance, indicating an obvious tendency that nearby cells are better correlated than the remote ones. Noteworthy, the values of correlations are very high, even at large intercellular distances. This, however, is not a consequence of cell-to-cell interactions, but is rather associated with the fact that several cells respond to the stimulation simultaneously, i.e. in a short time interval, irrespective of both, the way of intercellular communication and their locations. Yet, the obviously decreasing trend in Fig 4B puts forward the existence of intercellular communication mechanisms, which give rise to synchronized responses between adjacent LECs. These issues are addressed in more details in the next sections.

### Analysis of activations and deactivations of human LCs

**Fig 5:**
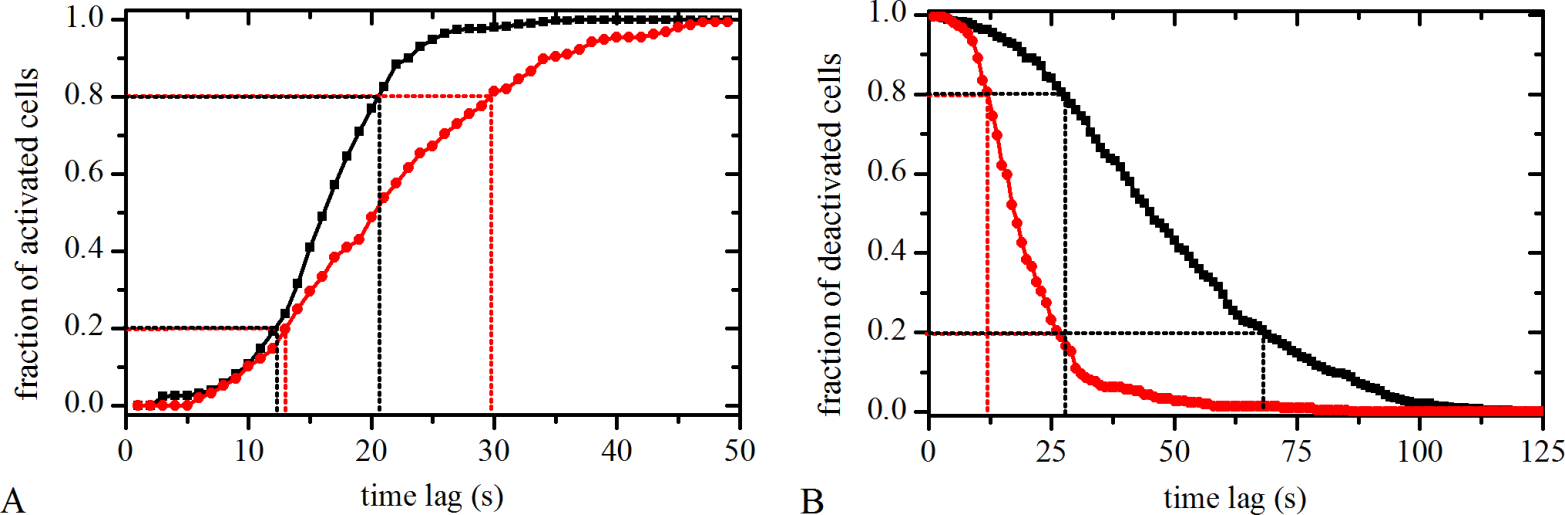
Temporal characterization of LECs activations/deactivations in response to ACh. **A** The fraction of activated LECs as a function of the time delays counted from the moment of activation of the first cell on. The diagram shows dependencies for two different LCs (red and black curves). **B** The fraction of deactivated LECs as a function of the time delays counted from the moment of deactivation of the first cell on. The diagram shows dependencies for two different LCs (red and black curves). Dotted lines indicate the intervals, which served for the calculation of the activation and deactivation speeds in a given LC.

After stimulation with ACh the response times, *t*_res_, characterizing the onsets of [Ca^2+^]_i_ increases in individual LECs, varied significantly from cell to cell. In order to characterize the responsiveness of a given LC we show in Fig 5A the fraction of activated LECs as a function of the time delay with regard to the first responding cells. The examples of two typical LCs that differ in their speeds of activation are presented in Fig 5 (red and black curves). Furthermore, we perform a similar analysis for the characterization of deactivations. When the [Ca^2+^]_i_ in a given LEC falls below 50% of its maximal value (at time *t*_half,*i*_), the cell is considered as deactivated. In Fig 5B we show the temporal evolution of deactivations in two different LCs. The time delay is counted from the *t*_half,*i*_ of the first deactivated cell on. For the description of the responsiveness with a single parameter, which enables a straightforward comparison between different LCs, we calculate the activation speed, *S*_A_, and the deactivation speed, *S*_DA_, which are defined by the slopes between 20/80% and 80/20% of activated/deactivated cells, respectively (see dotted lines in Fig 5).

**Fig 6:**
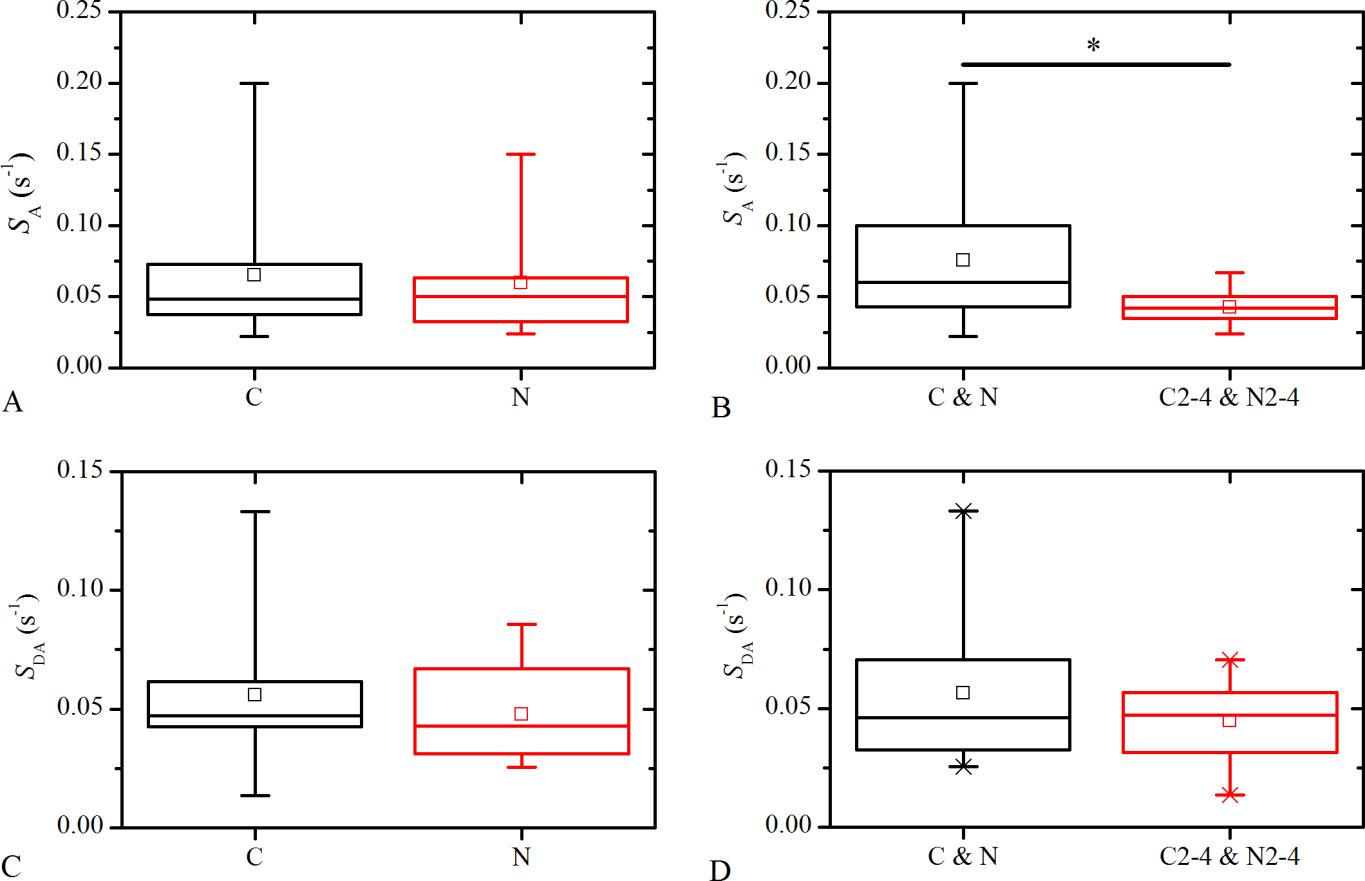
Analysis of the activation speeds, *S*_A_, and the deactivation speeds, *S*_DA_, of the anterior LCs. The comparison between the activation speeds, *S*_A_, (**A** and **B**) as well as between the deactivation speeds, *S*_DA_, (**C** and **D**) for: cortical, C, and nuclear, N, cataract LCs (**A, C** respectively), as well as, mild, N & C, and moderate to severe, C2-4&N2-4, cataract LCs (**B, D,** respectively); The asterisk in panel **B** indicates that the ANOVA test confirmed a statistical significant difference in the activation speeds, *S*_A_, when comparing the LCs with regard to the degree of the cataract. In all other comparisons no significant differences could be detected. Box charts are defined the same as in Fig 2.

The box charts in Fig 6 display the comparison of the activation speeds, *S*_A_, Fig 6A and 6B, and deactivation speeds, *S*_DA_, Fig 6C and 6D, with respect to the type and the degree of the cataract. It can be observed that in all cases the values span over rather broad intervals, thereby indicating high variability in *S*_A_ and *S*_DA_. Despite high interindividual variability, a statistically significant difference between activation speeds of mild, C&N, and moderate or severe, C2-4&N2-4, cataract LCs is detected indicating that the LCs with a smaller degree of cataract exhibit a faster global response to stimulation. On the other hand, the differences in the activation or deactivation speeds with regard to the type of the cataract were not found statistically significant. Moreover, we verified if the activation and deactivation speeds depend on spontaneous activity. Results in S3 Fig show that spontaneous activity of LECs does not have a significant impact on the activation or deactivation speeds of the whole LC. Apparently, the presence of spontaneously active cells significantly influences only the average activation times of individual cells, Δ*t*_act_, (see Fig 3 and S1 Fig), whereas on the other hand the responsiveness of the whole LC is not affected by the presence of spontaneously active LECs.

### Spatio-temporal grouping of human LECs

**Fig 7:**
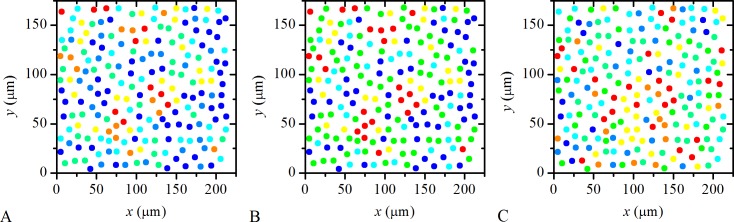
Visualization of the spatio-temporal organization of LECs activity in a typical human LC. **A** Color coded values of response times, *t*_res_, of individual LECs in a particular LC. Blue color denotes the time-delay of 0 s and red color denotes the time-delay of 8 s or more after the first responders. Light blue, green, yellow and orange colored LECs respond within 0 and 8 s with a time-step of 2 s, progressively. **B** Color coded values of activation times, Δ*t*_act_, for individual LECs in the same LC as in **A**. Blue color denotes Δ*t*_act_ of 10 s or less and the red color denotes Δ*t*_act_ of 22 s or more. Light blue, green, yellow and orange colors of LECs denote Δ*t*_act_ between 10 and 22 s with a time-step of 3 s, progressively. **C** Color coded values of deactivation times, Δ*t*_deact_, for individual LECs in the same LC. Blue color denotes Δ*t*_deact_ of 12 s or less and red color denotes Δ*t*_deact_ of 24 s or more. Light blue, green, yellow and orange colors of LECs denote Δ*t*_deact_ between 10 and 22 s with a time-step of 3 s, progressively. A visual inspection of the figure indicates that a tendency of nearby cells being activated simultaneously and having similar signal duration exists, as localized subgroups of cells with the same color can be identified in all three panels.

Next we focus on the spatio-temporal organization of LECs activity. For this purpose we color-code the values of the times at which LECs responded to Ach, *t*_res_, as well as the values of the activation times, Δ*t*_act_ = *t*_max_ − *t*_res_, and, the deactivation times, Δ*t*_deact_ = *t*_half_ − *t*_max_, of individual LECs (see Fig 1 & Materials and Methods). Fig 7 illustrates the results. It can be noticed that several localized subgroups of LECs exist, of which particular times of interest overlap. The fact that neighboring LECs have the same color indicates a tendency of nearby cells being activated simultaneously (Fig 7A) and having similar activation (Fig 7B) as well as deactivation times (Fig 7C).

**Fig 8:**
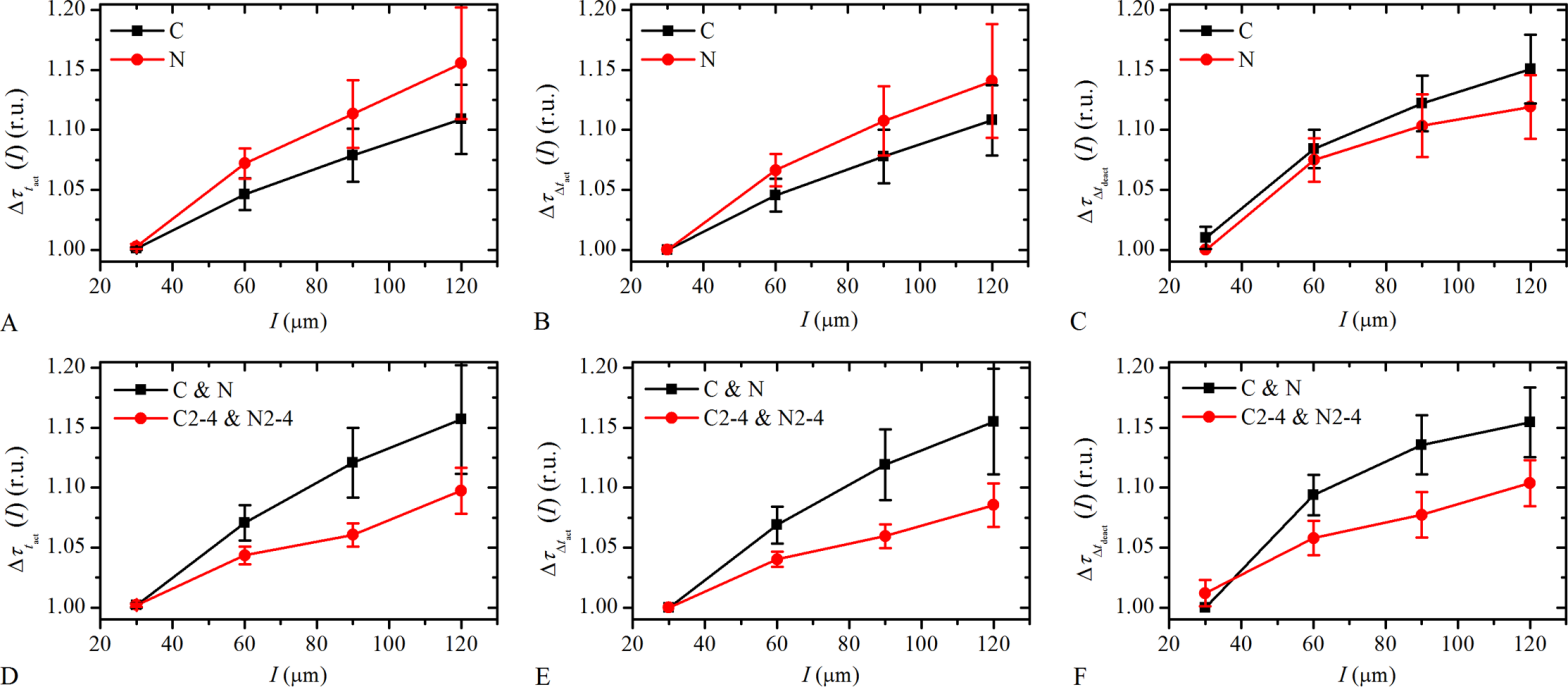
Quantification of the spatio-temporal grouping of LECs. **A-C** Differences in the response times, $\Delta \tau_{t_{\text{res}}}\left(I\right)$, the activation times, $\Delta \tau_{\Delta t_{\text{act}}}\left(I\right)$, and the deactivation times, $\Delta \tau_{\Delta t_{\text{deact}}}\left(I\right)$, as a function of the distance, *I*, with respect to the type of the cataract. **D-F** The same analysis of the spatio-temporal grouping with respect to the degree of cataracts. The temporal differences in all cataracts were normalized with the smallest identified value in order to overpass the variability in absolute values of signaling times in different LCs.

To quantify the grouping of cells with respect to their simultaneity of activations, *t*_res_, and the similarity in their activation and deactivation times, Δ*t*_act_ and Δ*t*_deact_, respectively, as observed in Fig 7, we calculate the average temporal difference within a given distance interval, Δ*I*, for all cell pairs, Δ*τ*_*x*_(*I*) (see Materials and Methods). The subscript *x* refers to different times of interest: $\Delta \tau_{t_{\text{res}}}\left(I\right)$ represents the differences in response times, $t_{\text{res}};\;\Delta \tau_{\Delta t_{\text{act}}}\left(I\right)$ refers to the differences in activation times, Δ*t*_act_; and $\Delta \tau_{\Delta t_{\text{deact}}}\left(I\right)$ refers to the differences in deactivation times, Δ*t*_deact_. The results are presented in Fig 8. In all cases Δ*τ*_*x*_(*I*) is found to increase with the distance, *I*. This demonstrates that LECs are not arranged randomly with respect to their activity, but they tend to form clusters with simultaneous activations or similar signal durations. However, different slopes of the curves can be noticed, which indicates the differences in the extent of the spatio-temporal grouping. Evaluation of the results with respect of the type of the cataract (Fig 8A–8C) reveals that the observed differences are within the order of the dispersion of the results. Therefore, we cannot make a distinction regarding the extent of grouping in C and N cataracts LCs. This is mostly due to a rather high level of variability. On the other hand, a comparison regarding the degree of the cataract (Fig 8D–8F) reveals that the slopes of the curves are substantially smaller in more developed cataracts. Apparently, the tendency of nearby cells having similar characteristics regarding their times of responses or the shapes of [Ca^2+^]_i_ signals decreases with the increasing levels of pathology.

### Network-based characterization of intercellular communication between LECs

**Fig 9:**
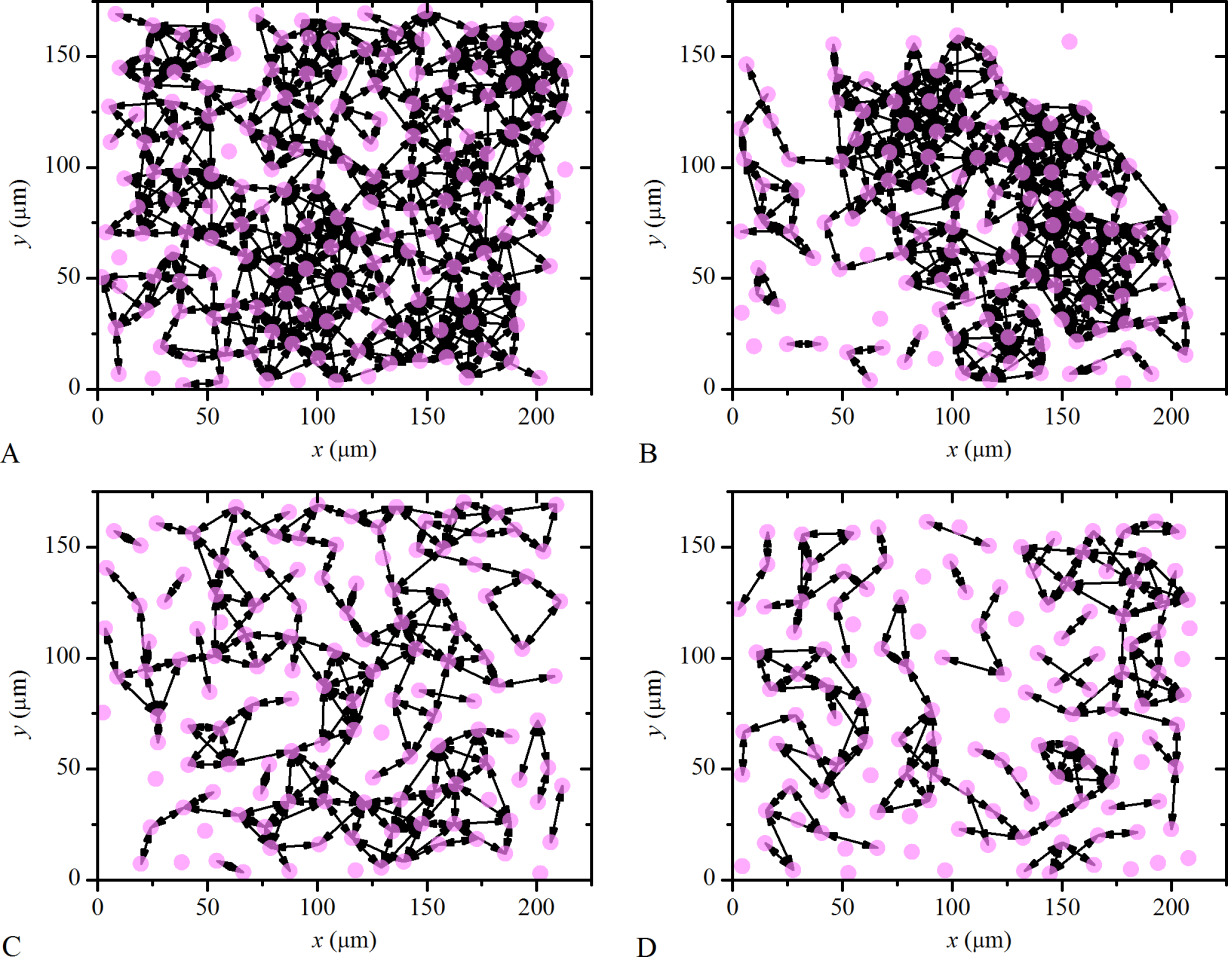
Characteristic networks of human LECs in four different LCs associated with different cataract types and degrees. **A** Mild cortical cataract, C, **B** Mild nuclear cataract, N, **C** Moderate cortical cataract, C2, and **D** Moderate nuclear cataract, N2. Evidently, both mild cataract LCs (C and N) represent quite densely connected and integrated networks, whereas on the other hand, the networks of both moderate cataract LCs (C2 and N2) appear to be much more sparsely connected and segregated.

To assess the nature of intercellular communication between LECs in more details, we use the theoretical framework, which was primarily developed in the field of complex networks. In particular, we construct the so-called activation networks on the basis of simultaneous activations of LECs (see Materials and Methods). A spatial constraint was introduced in order to exclude the existence of the connections between remote cells, which accidentally respond to ACh simultaneously (see Fig 7A) and not as a consequence of intercellular communication. Such example would be the propagation of Ca^2+^ waves. Accordingly, only the cells being located less than 30 μm apart can be considered as directly connected. The extracted networks can be subsequently directly used for the evaluation of the features of intercellular communication. Four typical lens epithelium networks corresponding to different types and degrees of the cataract are shown in Fig 9. There, one can observe that both LCs, associated with mild cataracts (C and N) (Fig 9A and 9B, respectively), result in much denser and more interconnected networks than in the case of lens epithelium associated with more developed cataracts (C2 and N2) (Fig 9C and 9D). On the other hand, visual inspection of the networks does not indicate any obvious differences between the LCs associated with different types of cataract (C or N).

**Fig 10:**
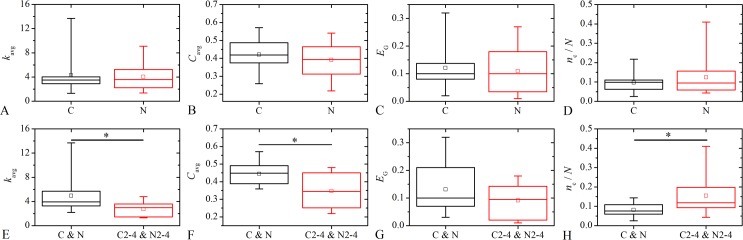
Characterization of the intercellular communication networks of LECs. Box charts show different network’s metrics of the LCs with respect to the type (**A-D**) and the degree (**E-H**) of the cataract. Network’s metrics presented on the panels **A-H** are: the average network’s degree, *k*_avg_ (**A** and **E**); the average clustering coefficient, *C*_avg_ (**B** and **F**); the global efficiency, *E*_G_(**C** and **G**); and, the relative number of communities (subcompartments), *n*_c,*i*_/*N*_*i*_ (**D** and **H**). Significant differences obtained after one-way ANOVA tests are indicated by asterisks. Box charts are defined the same as in previous figures.

To quantify the properties of the activation networks and to compare their characteristics with respect to the type or the degree of the cataract, we calculate several network metrics (see Materials and Methods). We calculate the network’s average degree, *k*_avg_, which reflects the level of connectedness within the tissue. The global efficiency, *E*_G_, reflects the network’s functional integration such as the traffic capacity. The next measure is the average clustering coefficient, *C*_avg_, that characterizes the cliquishness of a network by indicating how concentrated the connections around a typical cell are. Higher values of *C*_avg_ correspond to clustered connections around individual LECs. Finally, we compute the relative number of communities, *n*_c,*i*_/*N*_*i*_, that is a measure of the network’s segregation. If in the *i*-th LC there are relatively many different communities, this indicates that the network is very compartmentalized and segregated. Results are presented in Fig 10. In the upper row (Fig 10A–10D) the networks are compared with respect to the type of the cataract, whereas in the lower row (Fig 10E–10H) the comparison with respect to the degree of the cataract is shown. Evidently, all network measures span over rather wide intervals, thereby indicating that the intercellular communication between LECs is subjected to high levels of inter-capsule variability, as well. Notably, none of the comparisons could confirm significant differences in the lens epithelial networks with regard to the type of the cataract. In case of C and N cataracts the mean values and the dispersion were quite similar. On the other hand, significant differences were obtained by comparing the average network degree, the average network clustering and the fraction of communities in the networks regarding the cataract degree. The average network degree and the average network clustering were found bigger and the fraction of communities in networks was found smaller in mild cataracts. Considering the cataract degree, only the differences in the global efficiency of the networks were not found to be significantly different, though the average value in mild cataracts is higher than in more developed stages. These results indicate that the intercellular communication between LECs is affected by the severity of the cataract. Thus, in more developed cataracts networks are sparser and more segregated.

## Discussion

In the present paper we analyzed the intra- and inter-cellular Ca^2+^ signaling patterns in LECs. We were particularly interested in the comparison of the Ca^2+^ signaling characteristics with regard to the type of the cataract and the degree of cataract progression. With regard to the cataract type, C and N, we detected no significant differences in any herein examined aspect of Ca^2+^ signaling. A rather high inter-capsule variability in [Ca^2+^]_i_ responses was present. Noteworthy, Gupta et al. [5] also reported that [Ca^2+^]_i_ levels are always found high in LECs, irrespective of the type of the cataract. This might be an indication of an impaired Ca^2+^ homeostasis in both types of cataracts. Intracellular Ca^2+^ homeostasis might be broken down possibly due to Ca^2+^ influxes exceeding the ability of LECs to remove [Ca^2+^]_i_ from the cytosol. It is not clear, whether this is due to upregulated Ca^2+^ influx, impaired ability to remove excess [Ca^2+^]_i_ or due to a combination of both processes. This is the field to be studied further.

Since no significant differences were observed between C and N cataracts, we compared the Ca^2+^ signaling in LECs associated with mild, C&N, and moderate to severe, C2-4&N2-4, cataracts, irrespective of the type of cataract. Again, no significant differences were detected by comparing the Ca^2+^ signal characteristics in individual LECs. On the other hand, significant differences were observed on the multicellular level. First, cells associated with less developed cataracts were found to have better orchestrated and faster global response to stimulation by agonist ACh. They have higher activation speeds meaning that LECs are activated faster and more simultaneously once the first cell is activated. This could be related with the responsiveness of the receptors, which is potentially decreased in more developed cataracts. On the other hand, there are no differences in the activation and the deactivation times of individual cells either with regard to the type or the degree of the cataract, suggesting that the intracellular processes involved both in activation and deactivation phases of [Ca^2+^]_i_ response of more developed cataracts do not differ from those in less developed cataracts. The parameter *t*_half_ is likely related to the functionality of the Ca^2+^ removal mechanisms and our results suggest that they are not related with the degree of cataract. Second, we showed that with increasing levels of pathology, the tendency of nearby cells having similar characteristics regarding their times of activation or their durations of the Ca^2+^ signal, decreases. This implies the impairment of intercellular communication mechanisms and also suggests the existence of intercellular communication which ensures more synchronized responses between adjacent LECs in less developed cataract. To further address this issue we additionally described the connectivity patterns and signals propagation between LECs by means of graph-theoretical approaches. Our results show that the extracted intercellular networks are much sparser and more segregated in more developed than in mild cataracts. It appears evident that intercellular communication between LECs is affected by the severity of the cataract, which results in less integrated networks within more developed cataract stages. In mild cataracts the average network degree and the average network clustering were significantly larger, and the fraction of communities in the activation networks was smaller than in more developed cataracts. In other words, at higher degrees of cataract pathology, larger segregation occurs, resulting in less unified and less effective intercellular communication within the lens epithelium. The fact that LECs of C&N cataracts are better interlinked and their [Ca^2+^]_i_ response after ACh is faster and more synchronized than C2-4&N2-4 cataracts suggests that in more developed cataracts lens epithelium has functionally more damaged LECs, probably due to the loss of gap junctional coupling. This could have been expected, but we have clarified that by our present quantitative analysis.

We investigated also the extent and the role of spontaneous LECs activity. No significant correlation between the spontaneous activity and the type or the degree of cataract was detected. Rather large variability in the portion of spontaneously active LECs was observed. However, further analyses have revealed that individual spontaneously active LECs have on average higher activation times, Δ*t*_act_, in comparison to spontaneously non-active cells. This finding might suggest that spontaneously active LECs are damaged and have impaired intracellular mechanisms of Ca^2+^ increase regulation. Moreover, the larger activation times might also be related with the fact that after Ca^2+^ release from the endoplasmic reticulum in spontaneously active LECs a series of processes takes place, including the pumping of Ca^2+^ back into the internal stores. Consequently, the LECs might therefore respond with time delay due to the refractory period, in which they are less susceptible. We also showed that spontaneously active LECs usually form clusters, which together with the finding that spontaneously active LECs respond slower to ACh, suggest the existence of the regions of the human lens epithelium that are functionally damaged. The presence of spontaneously active cells influences significantly only the average activation times of individual cells, whereas the responsiveness of the whole LC is not affected. The fact that LECs’ spontaneous activity occurs irrespective of the cataract pathology might be an indication that these LECs’ functional changes are an additional disturbance of Ca^2+^ homeostasis, which could not be directly correlated with the type and/or the degree of cataract.

When functional impairment occurs in LECs, which is manifested by increased [Ca^2+^]_i_, LECs are not able to regulate Ca^2+^ homeostasis well anymore, and as a consequence, cataractogenesis is facilitated. Although the LEC is equipped with machinery to combat with cataractogenic insults, any alteration in the lens epithelium may proceed further in the remaining part of the lens and may lead to cataract [37]. The proper maintenance of Ca^2+^ levels by regulating the activity of Ca^2+^- pumps and Ca^2+^-channels as well as the inhibition of Ca^2+^-dependent enzymes is necessary for its homeostasis and can help in prevention of cataract [6]. LECs possess a multitude of mechanisms such as buffers, pumps and exchangers to keep [Ca^2+^]_i_ low as well as the mechanisms responsible for its increase [6]. Among the latter there are various Ca^2+^ channels across the plasma membrane as well as the IP_3_-gated channels and ryanodine receptor channels located in the ER.

In healthy LECs intracellular Ca^2+^ is strongly regulated and its concentration in the cytoplasm is several orders of magnitude less than in the extracellular environment. But Ca^2+^ levels are always found high in cataractous LECs, irrespective of the type of cataract [5]. Total lens Ca^2+^ is greatly elevated in human cortical cataract [38], as it is in most animal models for cataract [2]. On the other hand, pure nuclear cataracts have normal internal Ca^2+^ ionic content [38]. The monolayer of epithelium accounts for a miniscule fraction of the lens mass since there are around 2500 layers of fiber cells in the adult human lens [39]. Therefore, the fact that there is high Ca^2+^ level in LECs in N cataract is not in opposition to the finding that the pure N cataracts have a normal internal Ca^2+^ content. From the other side, altered Ca^2+^ homeostasis and Ca^2+^ signaling in LECs coincide with the increase in Ca^2+^ in the cortical lens region in the case of C cataract and not the N cataract.

In the context of LECs, previous studies have pointed out the importance of cell-to-cell communication among LECs and showed that inhibition of specific connexins that form the gap junction channels leaded to lens opacification [40–42]. Cx43 was identified as the major connexin protein responsible for the gap junctional communication between LECs [43], and Cx46 and Cx50 are the two most abundant gap junction proteins in lens fiber cells [44,45]. Mutations or “knock-out” of any of these genes resulted in cataract development in humans or animals (for review see [46]). Cx43 hemichannels and gap junctions have largely been described and studied also in non-lens cell types, where it was explicitly shown that Cx43-related gap junctions are gated by a Ca^2+^-calmodulin (Ca^2+^-CaM)-dependent mechanism [47]. In the LECs of sheep two mechanisms of modulating gap junctional coupling have been proposed. CaM-dependent inhibition of lens gap junctional coupling was evident with sustained, micro molar levels of intracellular Ca^2+^, whereas agonist-induced PKC activation resulted in a transient decrease in lens gap junctional coupling [48]. Since gap junction coupling allows passage of many different molecules, including signaling molecules and ions such as Ca^2+^ from cell to cell, it was suggested that gap junctional uncoupling and reduced cell-to-cell communication in LECs would be expected to limit further cell damage in a tissue [48]. It is suggested that calmodulin (CaM) -dependent closure of gap junctions might be a protective mechanism when there is a sustained elevation of Ca^2+^ such as in case of cataract [48].

In the cataractogenesis, intracellular overload with Ca^2+^ in LECs possibly triggers a series of events such as activation of Ca^2+^-dependent enzymes, irreversible breakdown of important structural proteins, and, finally cell death [5]. On the other hand, the death of LECs leads to rearrangement of LECs, which may further lead to uncoupling of cells, which is vital for the maintenance of the lens’ transparency [2]. It was shown that such uncoupling of cells and the breakdown of LECs intercellular connectivity causes dysfunction of active transport of electrolytes, causing passive inward movement of water and the progression of cortical cataract development [49].

Although cataract formation is mostly considered to be a multi-factorial disease, oxidative stress might be one of the leading causes for both N and C cataracts [50]. One view is that the most important initial mechanism for the progression of age-related cataract is oxidative damage in the range of fiber-cell membranes of the lens, and also lens proteins, due to free radicals [51,52]. On the other hand, several alterations in LECs can also contribute to the development of cataracts [53]. Because of their superficial location, LECs are the first lens cells being exposed to various insults that contribute to the cataract formation. Cataract due to UV radiation is initiated by the damage to the epithelium that involves a change in membrane permeability leading to the loss of homeostasis of ions within the lens [54]. Generation of reactive oxygen species in LECs may also influence the development of cataract. LEC membrane SH groups were found to be important in the regulation of ions as well as being targets of hydrogen peroxide [55]. Since LECs are nucleated and metabolically active, they are the best equipped lens cells to defend and protect the rest of the organ from these noxious stimuli. It is likely that the gap junction channels and the hemichannels formed by connexins in the lens epithelium may assist with tissue protection, and they may also contribute to the cell injury [53]. It was also shown that apoptosis of LECs is associated with non-congenital cataract formation [56]. Both in vitro and in vivo studies have shown that treatment of adult lens with stress factors induces apoptosis of LECs, which is followed by cataractogenesis [57]. Both types of cells, LECs and fiber cells, can thus be considered as the location of pathological processes, albeit with different. In LECs, the impaired function of transport results in a water penetration into the fiber cells, whereas in the case of the primary impairment of the fiber cells the prevalent mechanism is the denaturation process in the crystallines. According to the applied methodology, our aim was to study calcium dynamics in LECs referring to both types of cataracts, those in which LECs and those in which fiber cells are expected to be primarily affected.

As we have shown previously, the human LC preparation obtained during the cataract surgery represents a reliable system for studying cellular processes associated with cataract formation [32–34]. The main advantage of the human anterior LC preparation consisting of the monolayer of anterior LECs lying on the basal lamina is, compared to cultures of cells, its preservation of relatively natural conditions. Previously, mechanical stimulations was used to induce a rise in [Ca^2+^]_i_ in cultured bovine LECs [58]. Such stimulation of a single cell within a confluent layer was shown to initiate cell-to-cell Ca^2+^ signaling. However, in human LC preparation, the LECs remain connected to the neighboring LECs and to the underlying basement membrane, thereby ensuring the examination of Ca^2+^ signaling with preserved intercellular communication mechanisms. But, we have to stress that the LECs in our study were not in contact with the fiber cells due to cataract surgery extraction protocol. Therefore the cell-to-cell interactions were not as *in vivo*, as the influence of interactions of LECs with fiber cells is not considered. Nevertheless, the examined LC preparation made the investigation of different aspects of Ca^2+^ signaling within and among LECs available. Even more, our study revealed modifications in the intercellular communication abilities with regard to the level of pathology. These findings suggest that the cataract progression entails modifications of gap junctional communication, which opens some new issues that will need to be addressed during future efforts within this field of research, especially in terms of intercellular interactions and also the interconnectivity between LECs and fiber cells.

## Materials and Methods

### Ethics statement

The research followed the tenets of the Declaration of Helsinki. The study was approved by the National Medical Ethics Committee of the Republic of Slovenia and all patients signed informed consent before the operation.

### Lens capsules preparation

Experiments were done on the anterior LC preparations consisting of the monolayer of LECs attached to the basement membrane, i.e. the capsule matrix. The LCs were obtained routinely during cataract surgery performed at the Eye Hospital, University Medical Centre (UMC), Ljubljana, Slovenia. The central LECs were studied, from the approximately 5–5.5 mm circles of the central anterior LCs that were carefully removed by continuous curvilinear capsulorhexis. Altogether 25 LCs were used. The age of capsule donors was from 41 to 88 with the average being 72 years. After the surgery, each LC was stored in Minimal Essential Medium Eagle (MEM; Sigma), with added heat inactivated newborn calf serum, and transported to the laboratory. Until utilization the LCs were kept in a CO_2_ incubator (Innova CO-48; New Brunswick Scientific, USA) at 37°C and 5% CO_2_. The LCs were loaded with the AM ester of Fura-2 (Fura-2 AM; Invitrogen–Molecular Probes, USA). For loading Fura-2 AM in DMSO was suspended in 3 ml MEM to a final concentration of 2μM. The loading was done in the incubator at 37°C for 30 min. After loading, the LCs were washed twice for 7 min with MEM. LCs were then transferred to the plastic glass bottom Petri dishes (Mattek Corp., USA; 3.5 cm in diameter), filled with 3ml of the bath solution with (in mM): NaCl 131.8, KCl 5, MgCl2 2, NaH2PO4 0.5, NaHCO3 2, CaCl2 1.8, HEPES 10, glucose 10), pH 7.24. There they were immobilized by a harp-like grid, similar to the one used for experiments with small vertebrate brain slices [59], so that the addition of the agonist solution would not displace them. The grid also flattened the LC, which was necessary for the optical recording. The orientation of the LC was with the basement membrane to the bottom, so the agonist could easily diffuse to the cells without having to cross the barrier of the basement membrane. The Petri dish with the immobilized LC was then mounted on the inverted microscope Zeiss Axiovert S 100 (Carl Zeiss, Germany). We applied the agonist acetylcholine (ACh; Sigma, USA) in 10 μM concentration, which was enough to induce >90% maximal [Ca^2+^]_i_ response, according to the data by Collison et al. [10].

### Calcium imaging

Image acquisition was done with the 12-bit cooled CCD camera SensiCam (PCO Imaging, Germany). The software used for the acquisition was WinFluor (written by J. Dempster, University of Strathclyde, Glasgow, U.K.). Objectives used were: 40x/0,75 Plan-NeoFluar and 63x/1,25 oil Plan-NeoFluar (Zeiss, Germany). The light source used was XBO-75W (Zeiss, Germany) Xe arc lamp. The light intensity was attenuated when necessary with grey filters with optical densities 0.5, 1 and 2 (Chroma, USA). The excitation filters used, mounted on a Lambda LS-10 filterwheel (Sutter Instruments Co., USA), were 360 and 380 nm (Chroma, USA). Excitation with the 360 nm filter (close to the Fura-2 isosbestic point) allowed observation of the cells’ morphology and of the changes in the concentration of the dye, irrespective of changes in [Ca^2+^]_i_, while the 360/380 nm ratio allowed visualization of the Ca^2+^ concentration changes in the cytoplasm. Image acquisition, timing and filterwheel operation were all controlled by WinFluor software via a PCI6229 interface card (National Instruments, USA). The criteria for selecting the region for imaging were the presence of adherent cells and good cell morphology both assessed by observation of transilluminated and 360 nm fluorescencent images. Individual image frames were acquired every 500 ms resulting in frame cycles being 1 s long (2 wavelengths). All offline analyses were done with custom made scripts written in C/C++ and Python.

### Time series preparation and characterization

The [Ca^+2^]_i_ dynamics of individual LECs from the experimentally recorded data sets were measured off-line by exporting the 360/380 nm signal ratio for all ROIs using ImageJ software [60]. The signal ratio *u*_*i*_(*t*) represents the temporal dynamics of the *i*-th cell. Prior further analyses the extracted time series *u*_*i*_(*t*) were smoothed by applying an adjacency averaging procedure. The smoothed traces were computed as:

$$u_{i}\left(t\right)=\frac{1}{2m}\sum_{j=1}^{m}\;u_{i}\left(t-j\Delta t\right)+\frac{1}{2m}\sum_{j=1}^{m}\;u_{i}\left(t+j\Delta t\right),$$ (1)

whereby the value of *m* was chosen such, that it did not considerably disturbed the time series. In average around 5% of selected LECs in all LCs did not respond to ACh or their signals were distorted. These cells were excluded from further analyses. For the characterization of the [Ca^+2^]_i_ signaling in individual LECs we defined three characteristic times (see Fig 1B). The response time, *t*_res,*i*_, symbolizes the time at which the cells started to respond to the stimulation with ACh. The time at which the maximal amplitude of [Ca^+2^]_i_ was reached is labeled with *t*_max,*i*_. Lastly, the time *t*_half,*i*_ symbolizes the half decay time, i.e. time necessary for the 360/380 ratio to decay by 50% from the maximum towards its pre-stimulation value. These three times are then used to define the [Ca^+2^]_i_ signaling characteristics by means of the activation time, Δ*t*_act,*i*_ = *t*_max,*i*_ − *t*_res,*i*_ and the deactivation time, Δ*t*_deact,*i*_ = *t*_half,*i*_ − *t*_max,*i*_.

### Spatio-temporal coherency

For the characterization of dynamical correlations in [Ca^2+^]_i_ responses between LECs and their spatio-temporal aspects, we calculated the average correlation coefficient, *R*_avg_(*T*). To this purpose we first constructed the correlation matrix **R**(*T*), with the *ij*-th element *R*_*ij*_(*T*) representing the correlation coefficient among the recorded [Ca^+2^]_i_ signals *u*_*i*_(*t*) and *u*_*j*_(*t*) within a time window *T* ± Δ*t*. The correlation coefficient *R*_*ij*_(*T*) was thus computed as:

$$R_{ij}\left(T\right)=\frac{\sum_{T-\Delta t}^{T+\Delta t}\left(\bar{u}{\left(T\right)}_{i}-u_{i}\left(t\right)\right)\left(\bar{u}{\left(T\right)}_{j}-u_{j}\left(t\right)\right)}{S_{u_{i}}S_{u_{j}}},$$ (2)

where $\bar{u}{\left(T\right)}_{i}$ and $\bar{u}{\left(T\right)}_{j}$ stand for the average value of the signal in the *i*-th and *j*-th cell and their corresponding standard deviation are given by $S_{u_{i}}$ and $S_{u_{j}}$. All terms refer to a given time interval *t* ∈ [*T* − Δ*t*, *T* + Δ*t*]. The temporal average correlation in the given time window *R*_avg_(*T*) is then computed as:

$$R_{\text{avg}}\left(T\right)=\frac{1}{N\left(N-1\right)}\sum_{i\neq j}\;R_{ij}\left(T\right),$$ (3)

In our calculations we used a constant time interval, Δ*t* = 10 s, which was being slid along the time series with a time-step of 5 s.

Furthermore, to capture the spatial aspects of correlations between LECs, we computed the distance matrix **I** that entails the information about Euclidean distances between all pairs of cells. In particular, the *ij*-th element is defines as:

$$I_{ij}=\sqrt{{\left(x_{i}-x_{j}\right)}^{2}+{\left(y_{i}-y_{j}\right)}^{2}},$$ (4)

where (*x*_*i*_, *y*_*i*_) and (*x*_*j*_, *y*_*j*_) are the centers of ROIs of the *i*-th and *j*-th cell, respectively. We applied a spatial binning procedure to select all cell pairs, whose distance is within the interval *I*_*ij*_ ∈ [*I* – Δ*I*, *I* + Δ*I*], and calculate the corresponding average correlation coefficient *R*_avg_(*I*).

### Activation and deactivation grouping

To quantify the grouping of cells with respect to their simultaneous responses to stimulation, *t*_res,*i*_, and their activation/deactivation times, Δ*t*_act_/Δ*t*_deact_, we calculate the average temporal difference between these three characteristic times as a function of the distance *I*:

$$\Delta \tau_{x}\left(I\right)=\frac{1}{N}\sum_{i}\frac{1}{N\left(\Delta I\right)}\sum_{j\left(\Delta I\right)}\left|\tau_{i}-\tau_{j}\right|,$$ (5)

where is the number of all cells, *N*(Δ*I*) is the number of cells within the distance interval, Δ*I*, from the *i*-th cell, and the inner sum calculates corresponding time differences between the *i*-th and the *j*-th cell, whereby *j*(Δ*I*) runs through all the cells within a given distance interval Δ*I*. The subscript *x* refers to different times of interest with regard to $\tau :\;\Delta \tau_{t_{\text{res}}}\left(I\right)$ represents the differences in response times (*τ*_*i*_ and *τ*_*j*_ signify *t*_res_ of the *i*-th and *j*-th cell, respectively). Similarly, $\Delta \tau_{\Delta t_{\text{act}}}\left(I\right)$ refers to differences in activation times Δ*t*_act_ and $\Delta \tau_{\Delta t_{\text{deact}}}\left(I\right)$ to differences in deactivation times Δ*t*_deact_ (see Time series preparation and characterization). If Δ*τ*_*x*_(*I*) is an increasing function of *I*, the cells are not arranged randomly with respect to their intracellular signaling, but tend to form clusters with simultaneous responses or similar signal durations. To overpass the variability expressed by different average activation times in different LCs (see Fig 2), we normalize the values of Δ*τ*_*x*_(*I*) in individual LCs, so that the smallest value identified at any distance, is set to one. Afterwards, the values in different LCs are averaged, in accordance to the type or degree of the cataract.

### Formation and characterization of the activation network

For the characterization of the intercellular communication among LECs within a LC, we make use of graph theoretical approaches and construct a so-called activation network. The construction of the network is based on finding LECs pairs that responded to external stimulation with ACh within a narrow time interval. In particular, the *i*-th and *j*-th LECs are considered as connected, if they are simultaneously activated, i.e. |*t*_res,*i*_ − *t*_res,*j*_| ≤ 1 s. To prevent the creation of connections that are established due to simultaneous responses in different parts of the LC, we incorporate a spatial constrain that limits the edge formation only among cell pairs which are less than 30 μm apart. In this manner we extract the connectivity patterns that reflect the intercellular communication between LECs.

To quantify the extracted networks we calculate different networks metrics. The most fundamental one is the average degree of the network, *k*_avg_. This measure is defined as the averaged sum over all individual degrees, *k*_*i*_, i.e. number of connections of individual cells. The second measure is the global efficiency of the network, *E*_G_. This network feature is computed as averaged sum over the inverse shortest path lengths among individual cell pair. The shortest path length between the *i*-th and *j*-th *l*_*ij*_ cell equals the shortest geodetic distance between them or the smallest number of edges which separates them. Thereby, *E*_G_ characterizes the network’s functional integration that reflects the effectiveness of information exchange and synchronizability. The third implemented measure is the average clustering coefficient, *C*_avg_, which quantifies the level of clustered connectivity and functional segregation. To compute this network feature we implemented the algorithm introduced by Watts & Strogats [61]. Initially we computed local cluster coefficients *C*_*i*_ as:

$$C_{i}=\frac{2n_{i}}{k_{i}\left(k_{i}-1\right)},$$ (6)

where *n*_*i*_ stands for the actual number of existing edges among nodes adjacent to the *i*-th node. The average clustering coefficient, *C*_avg_, is than defined as the averaged sum over all local cluster coefficients. Lastly, we also evaluate the segregation of the activation network by measuring the degree of sub-compartmentation. To this purpose we followed the algorithm introduced by Blondel et. al. [62]. Briefly, nodes are divided into sub-graphs or communities. By continuously rearranging the community structure of the network (i.e. changing the number of communities, changing the node members within a community, etc.), the algorithm maximizes a measure called modularity *Q*, which quantifies the strength of the realized division into communities. The process is repeated until changes in *Q* become negligible after successful alternations of the networks community structure. As a result we gain the information on the number of communities, *n*_c_. To quantify the degree of segregation in a given activation network of a LC we compute the ratio *n*_c,*i*_/*N*_*i*_ where *n*_c,*i*_ refers to the average number of detected communities and *N*_*i*_ to the total number of LECs in the *i*-th LC. The values of *n*_c,*i*_/*N*_*i*_ are bounded within the interval between 1/*N*_*i*_ and 1, whereby lower values indicate a weaker segregation of the network.

### Statistical analysis

To test if any significant differences exist among the extracted quantities between cells belonging to specific samples, we performed the one-way ANOVA with Bonferroni test. We used a predetermined upper limit of probability for statistical significance throughout this investigation with *P* ≤ 0.05. The analysis was performed using OriginPro 8.5 (OriginLab Corporation, Northampton, USA).

## Supporting Information

S1 Fig Analysis of Ca^2+^ signals in individual LECs with respect to spontaneous activity. Comparison of: **A** normalized individual activation times, [Δ*t*_act,*i*_]_norm_, and **B** deactivation times, [Δ*t*_deact,*i*_]_norm_, between spontaneously active and non-active cells. In the normalization process all values were divided by the average Δ*t*_act_ or Δ*t*_deact_ in the given LC in order to ensure a reliable comparison between cells in different LCs. LCs without spontaneously active cells were excluded from the analysis. Altogether 3391 spontaneously non-active and 506 spontaneously active cells were included in the analysis. The asterisk indicate statistically significant differences in activation times, whereas on the other hand significant differences in deactivation times with regard to spontaneous activity could not be detected. Values within individual groups are represented by means of the box charts diagrams in which the boxes determinate the 25th and 75th percentiles, the whiskers denote the 5th and 95th percentiles, the crosses stand for the minimal and maximal values, the line within the box signifies the median, and the small square stands for the average.
(TIF)

S2 Fig Spatio-temporal organization of spontaneously active LECs in a characteristic LC. **A** Positions of LECs with marked spontaneously active cells (black rings, 29 of total 200 cells) in the LC. Positions of 6 spontaneously active LECs, for which traces are presented in the B panel (colored black rings). **B** Temporal traces of the normalized [Ca^2+^]_i_ dynamics, *u*′(*t*), for 6 selected spontaneously active LECs, as indicated by the coloring in panel A. It can be observed that the majority of spontaneously active cells is organized into localized small groups that exhibit rather aligned spontaneous [Ca^2+^]_i_ responses.
(TIF)

S3 Fig Analysis of the activation speeds, *S*_A_, (A) and the deactivation speeds, *S*_DA_, (B) with regard to spontaneous activity. The one-way ANOVA test did not detect significant differences between the two groups of LCs, i.e. the group with spontaneously active LECs and the group without. The results indicate that the spontaneous activity does not have a significant impact on the activation, *S*_A_, and deactivation, *S*_DA,_ speeds of LCs. Values within individual groups are represented by means of the box charts diagrams in which boxes determine the 25th and 75th percentiles, the whiskers denote the minimal and the maximal values, the line within the box signifies the median, and the small square stands for the average.
(TIF)
