# Supplementary figures and images for: The Analysis of Intracellular and Intercellular Calcium Signaling in Human Anterior Lens Capsule Epithelial Cells with Regard to Different Types and Stages of the Cataract
Source: PLoS One. 2015 Dec 4;10(12):e0143781. doi: 10.1371/journal.pone.0143781 (PMC4670133; doi:10.1371/journal.pone.0143781)

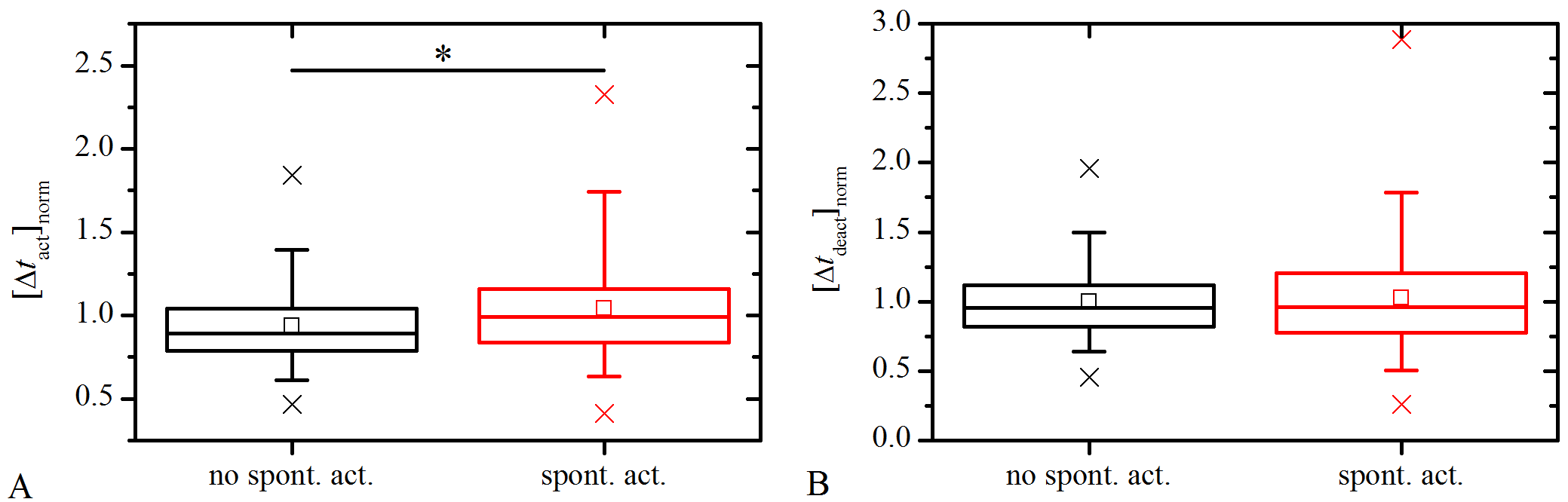

Supplement: S1 Fig — Comparison of: A normalized individual activation times, [Δt act,i]norm, and B deactivation times, [Δt deact,i]norm, between spontaneously active and non-active cells. In the normalization process all values were divided by the average Δt act or Δt deact in the given LC in order to ensure a reliable comparison between cells in different LCs. LCs without spontaneously active cells were excluded from the analysis. Altogether 3391 spontaneously non-active and 506 spontaneously active cells were included in the analysis. The asterisk indicate statistically significant differences in activation times, whereas on the other hand significant differences in deactivation times with regard to spontaneous activity could not be detected. Values within individual groups are represented by means of the box charts diagrams in which the boxes determinate the 25th and 75th percentiles, the whiskers denote the 5th and 95th percentiles, the crosses stand for the minimal and maximal values, the line within the box signifies the median, and the small square stands for the average. (TIF) [file pone.0143781.s001.tif]

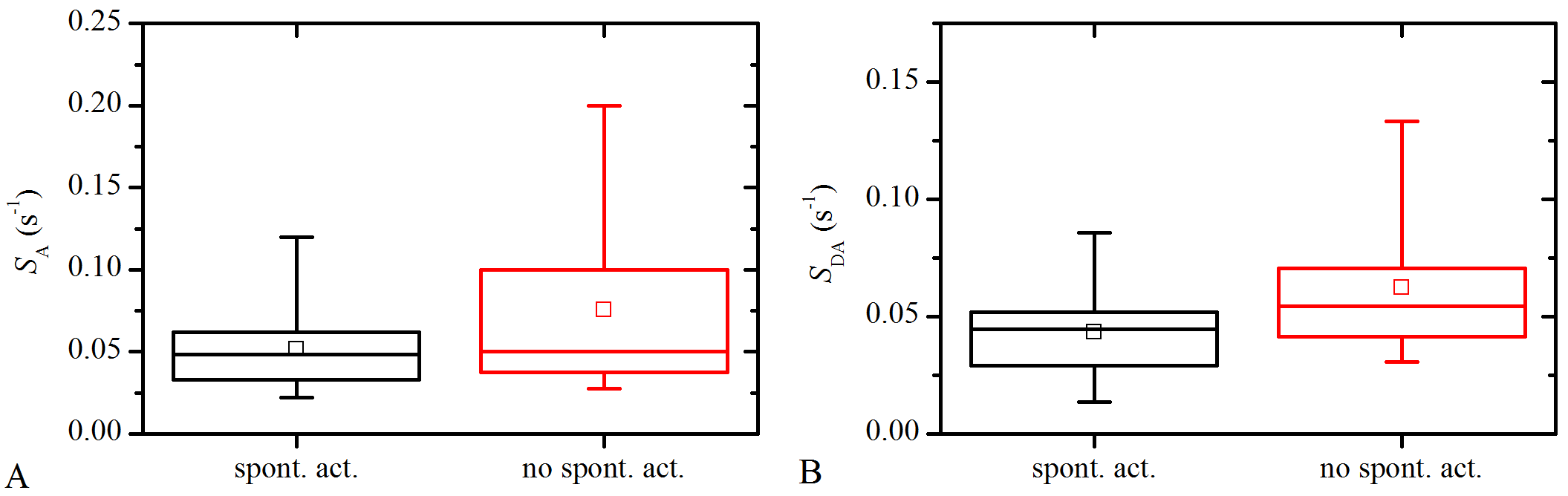

Supplement: S3 Fig — The one-way ANOVA test did not detect significant differences between the two groups of LCs, i.e. the group with spontaneously active LECs and the group without. The results indicate that the spontaneous activity does not have a significant impact on the activation, S A, and deactivation, S DA, speeds of LCs. Values within individual groups are represented by means of the box charts diagrams in which boxes determine the 25th and 75th percentiles, the whiskers denote the minimal and the maximal values, the line within the box signifies the median, and the small square stands for the average. (TIF) [file pone.0143781.s003.tif]
